# Microbial Activity Response to Solar Radiation across Contrasting Environmental Conditions in Salar de Huasco, Northern Chilean Altiplano

**DOI:** 10.3389/fmicb.2016.01857

**Published:** 2016-11-22

**Authors:** Klaudia L. Hernández, Beatriz Yannicelli, Lasse M. Olsen, Cristina Dorador, Eduardo J. Menschel, Verónica Molina, Francisco Remonsellez, Martha B. Hengst, Wade H. Jeffrey

**Affiliations:** ^1^Centro de Investigación Marina Quintay CIMARQ, Facultad de Ecología y Recursos Naturales, Universidad Andres BelloSantiago, Chile; ^2^Instituto de Ciencias Marinas y Limnológicas, Universidad Austral de ChileValdivia, Chile; ^3^Centro de Estudios Avanzados en Zonas AridasLa Serena, Chile; ^4^Facultad de Ciencias del Mar, Universidad Católica del NorteCoquimbo, Chile; ^5^Ecology and Sustainable Management of Oceanic Islands, Universidad Católica del Norte, CoquimboCoquimbo, Chile; ^6^Centro Universitario de la Región Este, Universidad de la RepúblicaRocha, Uruguay; ^7^Norwegian Polar InstituteTromsø, Norway; ^8^Laboratorio de Complejidad Microbiana y Ecología Funcional and Departamento de Biotecnología, Facultad de Ciencias del Mar y Recursos Biológicos, Universidad de AntofagastaAntofagasta, Chile; ^9^Centro de Biotecnología y BioingenieríaSantiago, Chile; ^10^Programa de Postgrado en Oceanografía, Departamento de Oceanografía, Universidad de ConcepciónConcepción, Chile; ^11^Centro de Investigación Dinámica de Ecosistemas Marinos de Altas Latitudes (FONDAP-IDEAL), Universidad Austral de ChileValdivia-Punta Arenas, Chile; ^12^Departamento de Biología, Observatorio de Ecología Microbiana, Facultad de Ciencias Naturales y Exactas, Universidad de Playa AnchaValparaíso, Chile; ^13^Laboratorio de Microbiología Aplicada y Extremófilos, Departamento de Ingeniería Química, Universidad Católica del NorteAntofagasta, Chile; ^14^Departamento de Ciencias Farmacéuticas, Facultad de Ciencias, Universidad Católica del NorteAntofagasta, Chile; ^15^Center for Environmental Diagnostics and Bioremediation, University of West Florida, PensacolaFL, USA

**Keywords:** extremophiles, Central Andes, Salar de Huasco, bacterial secondary production, solar radiation, light history, heterogeneous microbial production, environmental gradients

## Abstract

In high altitude environments, extreme levels of solar radiation and important differences of ionic concentrations over narrow spatial scales may modulate microbial activity. In Salar de Huasco, a high-altitude wetland in the Andean mountains, the high diversity of microbial communities has been characterized and associated with strong environmental variability. Communities that differed in light history and environmental conditions, such as nutrient concentrations and salinity from different spatial locations, were assessed for bacterial secondary production (BSP, ^3^H-leucine incorporation) response from short-term exposures to solar radiation. We sampled during austral spring seven stations categorized as: (a) source stations, with recently emerged groundwater (no-previous solar exposure); (b) stream running water stations; (c) stations connected to source waters but far downstream from source points; and (d) isolated ponds disconnected from ground sources or streams with a longer isolation and solar exposure history. Very high values of 0.25 μE m^-2^ s^-1^, 72 W m^-2^ and 12 W m^-2^ were measured for PAR, UVA, and UVB incident solar radiation, respectively. The environmental factors measured formed two groups of stations reflected by principal component analyses (near to groundwater sources and isolated systems) where isolated ponds had the highest BSP and microbial abundance (35 microalgae taxa, picoeukaryotes, nanoflagellates, and bacteria) plus higher salinities and PO_4_^3-^ concentrations. BSP short-term response (4 h) to solar radiation was measured by ^3^H-leucine incorporation under four different solar conditions: full sun, no UVB, PAR, and dark. Microbial communities established in waters with the longest surface exposure (e.g., isolated ponds) had the lowest BSP response to solar radiation treatments, and thus were likely best adapted to solar radiation exposure contrary to ground source waters. These results support our light history (solar exposure) hypothesis where the more isolated the community is from ground water sources, the better adapted it is to solar radiation. We suggest that factors other than solar radiation (e.g., salinity, PO_4_^3-^, NO_3_^-^) are also important in determining microbial productivity in heterogeneous environments such as the Salar de Huasco.

## Introduction

Biological communities located at high altitude endure extreme conditions such as high daily temperature variability and high incident solar radiation (Rothschild and Mancinelli, 2001; Catalán et al., 2006; Albarracín et al., 2015a,b). In addition, high altitude saline wetlands from the Andean Altiplano present contrasting mineral composition at small spatial scales (Acosta and Custodio, 2008; Risacher and Fritz, 2009; Uribe et al., 2015) that range from freshwater to hypersaline conditions, as well as from limiting to high nutrient concentrations (Dorador et al., 2008b, 2010). While biodiversity of higher trophic levels is low in extreme environments (Guerrero et al., 2013), there is evidence that lakes located above 2000 m (e.g., Tibet, Pyrenean, or Andean lakes) hold a large microbial diversity (Wang et al., 2011; Triadó-Margarit and Casamayor, 2012; Albarracín et al., 2015a,b) in association with environmental heterogeneity.

One of the variables that define high altitude systems as extreme environments for life is solar radiation (Rothschild and Mancinelli, 2001; Piacentini et al., 2003; Cordero et al., 2014; Albarracín et al., 2015a,b). Low zenith angle and high altitude result in incident solar radiation that would be detrimental for aquatic microbial productivity at sea level (Helbling et al., 2001; Sommaruga, 2001; Alonso-Saez et al., 2006; Santos et al., 2012) and could be enhanced by thin ozone and reflectance by clouds and salt (Lovengreen et al., 2005; Häder et al., 2015). Solar radiation (in the visible as well as UV range) can induce changes in heterotrophic bacteria community structure, growth and production directly or indirectly (Jeffrey et al., 2000). High levels of UV radiation are known to affect cell structure, function, and integrity (Häder et al., 2015). As a result, phototolerant or resistant microbial strains dominate in systems exposed to high solar radiation (Fernández-Zenoff et al., 2006; Flores et al., 2009; Paulino-Lima et al., 2013; Zenoff et al., 2014). In marine systems, bacterial secondary production (BSP) under solar radiation has been shown to depend on the solar exposure history (photobiological history) of the bacterioplankton community (Jeffrey et al., 1996a,b; Hernández et al., 2006). The detrimental effect of UV on community production from waters exposed to high solar radiation for long periods of time are significant, but lower than that of communities previously unexposed (Hernández et al., 2006; Santos et al., 2012). This is supported by observed differences in the presence of phototolerant strains between communities with different solar exposure histories (Fernández-Zenoff et al., 2006; Zenoff et al., 2014; Albarracín et al., 2016).

Although a few reports on primary production are available for high altitude Andean lakes, such as the deep oligotrophic Lago Titicaca (Lazzaro, 1981; Villafañe et al., 1999; Helbling et al., 2001), Lago Chungará and Laguna Negra (Cabrera and Montecino, 1987), and benthic primary production at Salar de Huasco (De la Fuente, 2014), to the best of our knowledge, no reports on BSP are available for high altitude northern Andean wetland systems. Nevertheless, it is reasonable to consider the importance of microbial productivity knowing that invertebrate and vertebrate populations are permanently supported by these systems (Márquez-García et al., 2009; Vila et al., 2013) and adapted microbial communities might show marginal detrimental responses to high solar radiation exposure.

In extreme environments, strong spatial and temporal heterogeneity of physico-chemical conditions promote a variety of niches (Albarracín et al., 2015a,b) affecting microbial production, diversity, and community structure within the same system (Rodríguez-Valera et al., 1985; Pedrós-Alió et al., 2000; Benlloch et al., 2002; Oren et al., 2009; Pollet et al., 2010; Wang et al., 2011). Species richness and BSP have been shown to have either insignificant (Wang et al., 2011) or negative (Benlloch et al., 2002; Gasol et al., 2004; Pollet et al., 2010) responses along positive salinity gradients. Freshwater to hypersaline conditions found at different location within a salt flat can lead to variation in microbial communities characterized by their tolerance/need of NaCl (Fernández-Zenoff et al., 2006; Flores et al., 2009; Paulino-Lima et al., 2013). Environmental spatial heterogeneity has been related to microbial abundance (e.g., bacteria, archaea, and microalgae) and biomass as well as to the development of highly diverse bacterial communities in water and sediment of high altitude systems (Dorador et al., 2003, 2008a,b, 2009, 2010, 2013; Márquez-García et al., 2009; Demergasso et al., 2010; Thiel et al., 2010; Vila et al., 2013). However, it is not yet possible to infer how the BSP of communities from extreme environments responds to strong environmental variability in a narrow spatial scale.

The high altitude saline wetland ecosystem of Salar de Huasco is formed by an intricate mosaic of different ground sources, streams, and shallow permanent and non-permanent ponds (Dorador et al., 2008a, 2010). During the dry season, the wetland receives an important input of low salinity groundwater from point sources (Acosta and Custodio, 2008) with no recent history of exposure to solar radiation. Chloride concentration (as a conservative tracer) in ponds and streams reflect the local balance of water loss (evaporation) and groundwater input (Acosta and Custodio, 2008) so high salinity levels mainly reflect surface evaporation and a longer history of solar radiation exposure. The dynamic configuration of Salar de Huasco provides an opportunity to study solar radiation effects on BSP under different salinity and nutrient concentrations. We hypothesized that the inhibitory effects of solar radiation on BSP are lower in waters with higher salinity (and therefore a longer history of solar exposure) than in those recently emerged and exposed (low salinity groundwater). To test this hypothesis, we: (i) quantified bacterial biomass and BSP in different water bodies in Salar de Huasco, (ii) determined BSP response to solar radiation spectra in selected stations, and (iii) characterized each site regarding physico-chemical (salinity, nutrients) and biological (microalgae composition, abundance, photosynthetic efficiency, chlorophyll *a*) conditions to establish their relationships with BSP measurements.

## Materials and Methods

### Study Area

**FIGURE 1:**
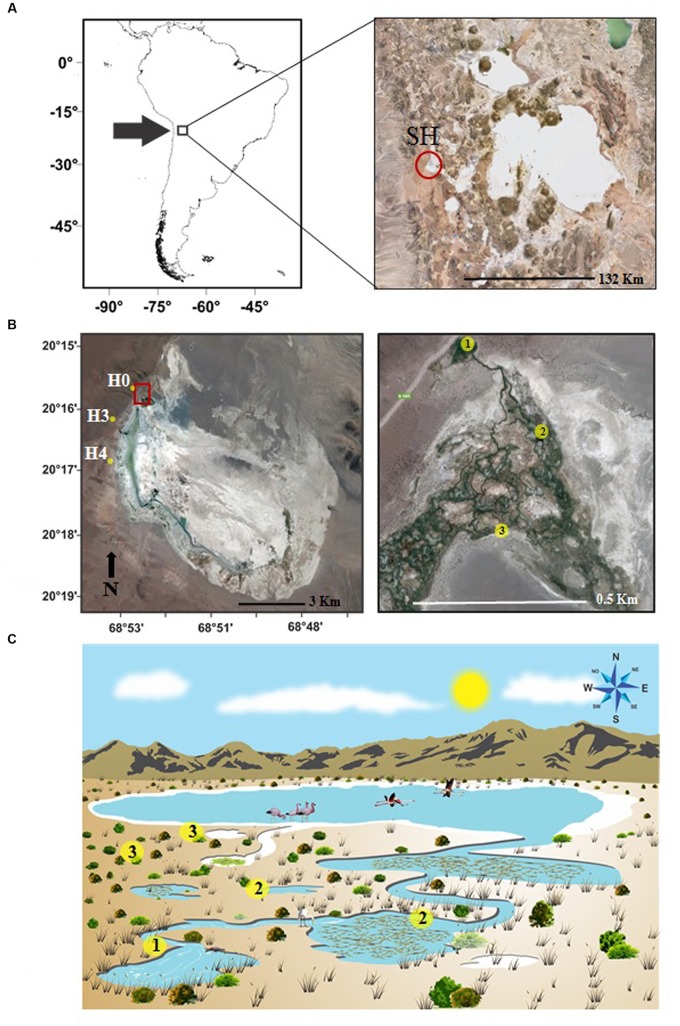
**(A)** Location of the study area at Salar de Huasco (SH), Chile. **(B)** Distribution of sampled stations along Salar de Huasco on previously described subsystems H0, H3, and H4 (Dorador et al., 2008b). Details of the intricate water flow system are shown as station types: (1) ground sources stations, with no previous solar exposure, (2) running water and connected ponds stations, and (3) isolated ponds. **(C)** Conceptual diagram of the Salar de Huasco suggested for the water flow connectivity and microbial communities distribution: ground sources H0 and H3 represented by yellow circle (1), close and connected downstream stations H3-RW and H4-CP with yellow circle (2), isolated ponds closer to the main shallow lake with H3-IP, H4-IP, H4-VMP represented by yellow circle (3).

Salar del Huasco (20.274°S, 68.883°W, 3800 m) is a saline wetland from the Tarapacá Region in northern Chile (**Figure 1A**) recognized as a Ramsar site by the Convention on Wetlands of International Importance (Ramsar Convention, 1996). Precipitation at Salar de Huasco is low and occurs over a short summer rainy season (Risacher et al., 1999; Risacher and Fritz, 2009; Sieland, 2014). Surface water flow (especially during the rainy season) and groundwater contribute to the maintenance of the lagoon, while evaporation of groundwater and surface water account for the only loss from the system (Uribe et al., 2015). The aquifers providing groundwater are re-filled seasonally after the percolation of precipitation. Infiltration due to surface characteristics and flatness of the relief is highly variable, forming a shallow but complex water system. Water flow varies abruptly in less than a kilometer of distance (**Figure 1B**). Several streams flowing from groundwater sources into a lagoon form water ponds with higher residence time which eventually become isolated due to evaporation (**Figure 1C**). The aquifer feeding groundwater-dependent streams shows a mineral composition similar to that of the crust and overlaying sediments which change mainly due to evaporation and water flow (Risacher et al., 1999; Acosta and Custodio, 2008; Risacher and Fritz, 2009; Sieland, 2014). Therefore, physico-chemical conditions within Salar de Huasco have been described as highly heterogeneous (Acosta and Custodio, 2008; Dorador et al., 2008a,b, 2009, 2013).

### Sampling Stations

Groundwater sources (and water bodies fed by them) northwest of the lagoon of Salar de Huasco have been previously characterized in terms of physico-chemical properties and microbial diversity (Magaritz et al., 1990; Dorador et al., 2003, 2008b, 2010; Acosta and Custodio, 2008; Vila et al., 2013). During November 15, 2013, we selected three of these subsystems (stations H0, H3, H4; Dorador et al., 2008b, 2010), and within them a total of seven stations were chosen considering site connectivity (distance and isolation) from the corresponding groundwater source (**Figures 1B,C**). Following these criteria, we expected to include sites with contrasting conditions regarding salinity and solar exposure history, with minima at the sources of groundwater and maxima at ponds isolated by evaporation.

With the previous considerations we sampled: (i) sources, with recently emerged groundwater and no-previous solar exposure, stations H0 and H3; (ii) streams and ponds with running water connected to sources (50 m downstream from sources, with recent solar exposure), stations stream running water (H3-RW), and H4 connected pond (H4-CP); (iii) isolated ponds disconnected from streams and sources (450 m from sources), with longer solar exposure, H4 isolated pond (H4-IP) and H3 isolated pond (H3-IP); and (iv) isolated pond disconnected from streams and sources (500 m from sources, with longer solar exposure), H4 isolated pond “Virgin Mary” (H4-VMP) (**Figure 1C**; **Supplementary Figure S1**).

### Solar Radiation Measurements

Incident solar radiation was monitored during the experiment using a GUV-511C radiometer (Biospherical Instruments). The GUV511 is a temperature-stabilized, multichannel radiometer that measures downwelling irradiance with moderately narrow bandwidth channels (near 10 nm) within the UVR (380, 340, 320, 305 nm), plus a broad band channel with full width at maximum of 300 nm for PAR (400–700 nm). Data points were recorded every minute between sunrise and sunset and processed using software provided by Biospherical Instruments. The total UVA (320–400 nm) and UVB (280–320 nm) irradiances were calculated by following Orce and Helbling (1997).

### Environmental Physico-Chemical Characterization

Temperature and conductivity were measured *in situ* at each sampling site with a Multiparameter pH/ISE/EC/DO/Turbidity Waterproof Meter with GPS option (Hanna Instruments, HI9829). Conductivity was converted to salinity (practical salinity units) considering the temperature of measurement using the Chemiasoft online calculator (http://www.chemiasoft.com/chemd/salinity-calculator) following Standard Methods for the Examination of Water and Wastewater, 20th edition (1999). Approximately 5 l of water were collected at dawn from each station on November 15, 2013. Subsamples were taken for different supporting parameters and the remaining water samples were kept submerged at H0 until water was distributed for the BSP incubation. Samples for nutrient determinations (two replicates) were collected from each sampling site for nitrate (NO_3_^-^), nitrite (NO_2_^-^), and phosphate (PO_4_^3-^) determinations soon after collection. Water was filtered through 0.7 μm pore-size (GF/F) filters and stored frozen in 15 ml polycarbonate tubes until analysis at the Laboratorio Biogeoquímica, Universidad de Concepción (Chile) using a Seal analytical AutoAnalyzer AA3 SEAL G-172-96 Rev 15 (Multitest MT 19 for NO_3_^-^ and NO_2_^-^) and G-297-03 Rev 3 (Multitest MT 19 for PO_4_^3-^). Silica (Si_2_O_3_^4-^) was determined as previously described (UNESCO, 1983).

### Chlorophyll *a*

Chlorophyll *a* was measured by filtering replicate 150 ml from each site onto Whatman GF/F filters, which were kept in the dark at -20°C until extraction in cold acetone (90%) for 24 h. The concentration of chlorophyll *a* was determined fluorometrically using a Turner Designs fluorometer (Model 10AU) previously calibrated against pure chlorophyll *a* (Sigma) following the method described by Holm-Hansen et al. (1965).

### *In vivo* Chlorophyll *a* Fluorescence Kinetics

Photosynthetic efficiency was measured with an AquaPen-C AP-C 100 fluorometer (Photon Systems Instruments). It was equipped with a blue and red light-emitting diode (LED), optically filtered to give light intensities of up to 3000 μmol photons m^-2^ s^-1^ to the samples on which it measures. Blue excitation light (455 nm) is for chlorophyll *a* excitation in eukaryote algae. Red-orange excitation light (620 nm) is for chlorophyll *a* excitation through phycobilin pigments in cyanobacteria samples (AquaPen manual, PSI). If the fluorescence signal was too weak due to low concentration of cells, the samples were concentrated by a filtration (Sartorius) with a 47 mm diameter polycarbonate filter of pore size 0.8 μm (Millipore) and a weak manual suction vacuum. The filtration was kept in motion to avoid cells settling on the filter. Three to four milliliters of each water sample was added to a plastic 1 cm cuvette and left in the dark inside the AquaPen instrument for 1 min before measurements to allow dark adaptation but not recovery from solar exposure. Fluorescence quantum yield (Qy) was measured by the saturation pulse method where the maximal fluorescence yield (*F*_m_) is measured by a flash with very high irradiance that saturates all the reaction centers in photosystem II (Cosgrove and Borowitzka, 2010). The minimum fluorescence (*F*_0_) was measured by a low measuring light that does not induce light reactions in photosystem II. The difference *F*_m_ -*F*_0_ is the variable fluorescence (*F*_v_). In a dark adapted sample (after 1–5 min), Qy = *F*v/*F*m. The Qy is a measure of how efficiently light is transferred into electron transport in the chloroplast membrane and thus into photosynthesis and can have values between 0 and 1, but values of 0.6–0.7 indicate good condition of photosystem II and are realistic maximum values for microalgae (Cosgrove and Borowitzka, 2010).

### Microalgae Abundance and Community Composition

One 250 ml sample of water from each site was taken and preserved with Lugol’s solution following Villafañe and Reid (1995). Samples were stored in the dark until analysis. Quantification of microalgae was performed using a light inverted microscope Nikon Eclipse TS 100 at 20× and 100× and equipped with an Olympus DP25 camera. A maximum of 50 ml was settled for low abundance samples. For high abundance samples, 5 ml were counted using the whole camera area following Utermöhl (1958). Abundance was expressed as number of Cells per liter by the formula: Cells/l = (N° cells × FC × 1000)/V. obs. where: N° cells = total number of cells quantified. FC = camera factor. V. obs = analyzed sample volume (ml). Diameter was measured for centric diatoms and length and width for pennate diatoms of different genera to estimate biovolume (BV). The BV of each taxon was calculated using the appropriate geometric formula or combination of geometric formulas that best represented observed cell shape (Edler, 1979). The microalgae identification was performed according to standard protocols (Cupp, 1943; Bourelly, 1970; Lazzaro, 1981; Liberman and Miranda, 1987; Wetzel and Likens, 1991; Parra and Bicudo, 1995; Tomas, 1997; Díaz and Maidana, 2005; Álvarez-Blanco et al., 2011; Blanco et al., 2013).

### Bacterial Secondary Production

We measured BSP following Smith and Azam (1992) with small modifications. From each sampling site, a subsample of 70 ml of water was spiked with sterile ^3^H-leucine (121 Ci mmol^-1^) to a final concentration of 10 nM. At the start of incubation, two 1 ml replicate samples of water from each sampling site were killed immediately with 5% (v/v) trichloroacetic acid (TCA) as a T = 0 control. The remaining labeled water from each site was distributed in 5 ml aliquots into 12 (60 ml) Whirlpack polyethylene bags to be incubated under four different solar conditions (Aas et al., 1996). Three bags were incubated without cover Full sun (PAB treatment), three bags were covered with Mylar 500D, 50% cut-off at 320 nm, i.e., no UVB (PA treatment), three bags were incubated wrapped in Court guard, 50% cut-off at 400 nm, no UVR (PAR treatment) and the remaining three bags were covered in opaque black plastic (Dark).

Solar radiation incubations started at approximately 10:30 hours and lasted for 4 h. All Whirlpack bags were held *in situ* attached to a tray submerged on the stream below H0 to assure homogeneous temperatures across incubations (**Supplementary Figure S1**). At the end of the experiment, TCA was added to each bag to a final concentration of 5% (v/v). Triplicate 1 ml subsamples were then transferred from each bag to microcentrifuge tubes which were processed as described in Smith and Azam (1992).

Leucine incorporation was measured using a Packard Model 1600TR liquid scintillation counter. The counting efficiency was calculated from the non-quenched standard of ^3^H-toluene. BSP from leucine incorporation was calculated using a ratio of cellular carbon to protein of 0.86 and a fraction of leucine in protein of 0.073 (Simon and Azam, 1989) and was expressed as μg C l^-1^ h^-1^. Finally, the cell-specific incorporation rate of carbon was calculated as the quotient of estimated BSP to total bacterial abundance (BA) determined for each water sample and expressed as fg C cell^-1^ h^-1^ following Hernández et al. (2007). Percentage inhibition/enhancement of BSP by PAR, UVA, or UVB was calculated with respect to dark BSP as 100% (Hernández et al., 2006).

### Flow Cytometry

Two replicate 1.35 ml subsamples were taken for each site in cryovials and fixed with glutaraldehyde (2% final concentration) and immediately frozen in liquid nitrogen. Photosynthetic and non-photosynthetic prokaryotic picoplankton abundances were estimated based on 165 particles counts of subsamples (150 μl) previously stained with SYBR-Green I (Molecular Probes; Marie et al., 1997). Light scatter and fluorescence measurements of particles were made using a FACSCalibur^TM^ flow cytometer (Becton Dickinson) with MED flux 30 and 34 (μl/min) using Cytowin software at Universidad de Concepción facilities. The FACSCalibur BD had five PMTs based on size (FCS), rugosity (SSC), phycobilins (FL1), phycoerythrins (FL2) and chlorophyll (FL3). Several groups of picophytoplankton are reported including total abundance of picoeukaryotes (PE), bacterial (bacteria and archaea) and nanoflagellates (NA) as cells l^-1^.

### Statistical Analysis

In order to evaluate bio-physical environmental similarity between stations, we conducted ordination analysis considering salinity, NO_2_^-^, PO_4_^3-^, and NO_3_^-^ concentrations, chlorophyll *a* concentrations, and photosynthetic efficiency as Qy450 and Qy620. Principal component analysis (PCA) was conducted after data standardization (variables standardized to the maximum) using Primer 6 software. In order to evaluate the similarity of sampling sites based on microalgae community composition and abundance, we performed a classification analysis. Microalgae abundance data were fourth root transformed and the dissimilarity between stations was calculated as the Bray–Curtis dissimilarity index (Legendre and Legendre, 1998) to conduct group average clustering using Primer 6 software.

A covariance analysis was conducted on BSP response to solar radiation (fixed factor, four levels) with distance from source points as the covariate chosen to evaluate the interaction between type of site and BSP response to the different treatments. Two stations (H0 and H3) were at 0 m from point sources of groundwater. H3-RW and H4-CP were 50 m downstream from point sources, and isolated ponds were 450 and 500 m. The analysis considered the mean value of BSP measured for each site and treatment, disregarding/neglecting the within site variability to avoid pseudoreplication (Hurlbert, 2004). This study combines a manipulative approach to the response of microbial communities to solar radiation at Salar de Huasco, with the explicit consideration of spatial characteristics of water bodies at the scales of tens to hundreds of meters (manipulative-correlative study sensu; Hewitt et al., 2007).

After covariance for treatments and groups of stations at different distance from source was confirmed, BSP response to different solar radiation treatments (four levels) was compared for each station with one-way ANOVA. BSP was previously transformed (square root) to meet variance homogeneity assumptions when it was needed. *Post hoc* Tukey tests were conducted to identify the specific differences between BSP among solar radiation treatments after checking for the significance of the main factor as a whole (Statistica 7 software). When ANOVA assumptions were not met even after root (or logarithmic transformation), a non-parametric Kruskal–Wallis test was applied followed by the test of rank means (Statistica 7). Finally, a cluster analysis (group average clustering) was conducted on the Euclidean distance matrix between stations based on the standardized mean BSP per station and treatment. The analysis allowed the grouping of stations with the same pattern of response to the different solar treatments.

## Results

### Solar Radiation

**FIGURE 2:**
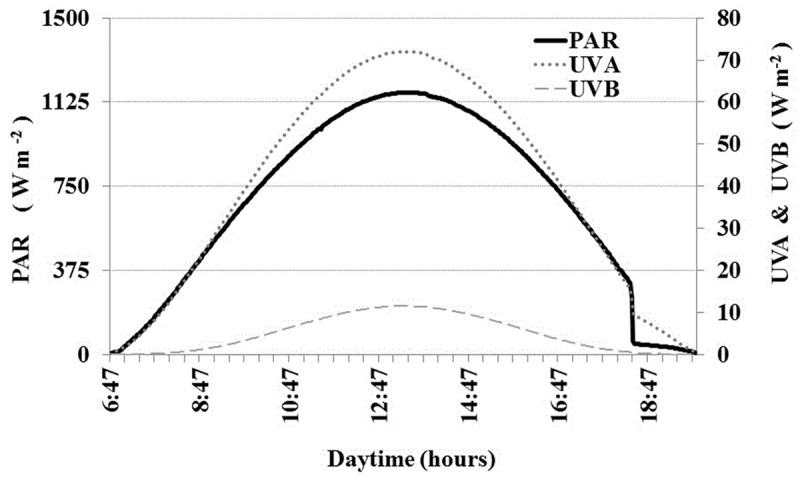
**Solar radiation diurnal cycle in Salar de Huasco during November 15, 2013 (radiometer GUV511C).** Continuous PAR (left *y*-axis), UVA and UVB (right *y*-axis). Incident irradiance was expressed as W m^-2^. Data break at approximately 18:00 was due to sunset behind the surrounding mountains.

During November 15, 2013, maximum irradiance reached 1168 W m^-2^ (0.25 μE cm^-2^ s^-2^) for PAR, 72 W m^-2^ for UVA and 12 W m^-2^ for UVB at 1:13 pm local time (**Figure 2**). The sky was cloudless and the solar day was approximately 12 h (from 6 am to 6 pm).

### Environmental (Physico-Chemical and Biological) Characterization

Salinity was below 1 across all sampled sites, except for H4-VMP, the only hypersaline site (49.1 PSU; **Table 1**). Among the other stations, sources and H3-RW were approximately 0.3 PSU while isolated and connected ponds were slightly saltier, about 0.55 PSU. While silica concentrations were above 40 mM at all sites, NO_3_^-^ concentration varied over several orders of magnitude (**Table 1**). Ponds showed no detectable to 0.1 μM concentrations (H4-VMP) and sources and the stream waters contained over 10 μM of nitrate NO_3_^-^. PO_4_^3-^ was also lower in ponds (except H4-VMP), while sources and the stream. In contrast, H4-VMP presented a PO_4_^3-^ peak concentration of 81.1 μM (**Table 1**).

**Table 1:** Physico-chemical characterization during sample collection at selected stations in Salar de Huasco during November 15, 2013.

Stations^∗^	Temp (°C)	Salinity (PSU)	NO_3_^-^ (μmol L^-1^)	NO_2_^-^ (μmol L^-1^)	PO_4_^3-^ (μmol L^-1^)	Si_2_O_3_^4-^.(mmol L^-1^)
H0	13	0.38	12.2	0.5	1.4	≥40
H3	13	0.3	11.6	0.2	1.0	≥40
H3-RW	15	0.29	11.3	0.3	0.97	≥40
H4-CP	0.1	0.56	BDL^∗∗^	0.3	0.8	≥40
H4-IP	0.3	0.6	BDL	0.3	0.9	≥40
H3-IP	5	0.55	BDL	0.3	0.5	≥40
H4-VMP	0.1	49.1	0.1	0.1	81.1	≥40


*Temp, temperature; PSU, salinity; NO_*3*_^-^, nitrate; NO_*2*_^-^, nitrite; PO_*4*_^*3*-^, phosphate; Si_*2*_O_*3*_^*4*-^, silicate.*

*^∗^Seven stations were selected as follows: (a) Ho source (H0); (b) H3 source (H3); (c) H3 stream running water (H3-RW); (d) H4 connected pond (H4-CP); (e) H4 isolated pond (H4-IP); (f) H3 isolated pond (H3-IP); (g) H4 isolated pond “Virgin Mary pond” (H4-VMP).*

*^∗∗^Values below detection limits (BDL).*

Chlorophyll *a* was lowest in source waters (0.3–1 μg l^-1^), higher in streams and ponds (from 3 to 4 μg l^-1^), and intermediate at H4-VMP (1.6 μg l^-1^; **Table 2**). Qy450 as photosynthetic efficiency for eukaryotes was higher in source and stream waters (above 0.43), and lower in ponds, except at H4-IP. Qy620 for Cyanobacteria was highest in H3-RW.

**Table 2:** Biological characterization of seven stations in Salar de Huasco during November 15, 2013.

Stations^∗^	BSP (μg C L^-1^ h^-1^)	BA (cells L^-1^)	PE (cells L^-1^)	NA (cells L^-1^)	MB (μg C L^-1^)	MA (cells L^-1^)	Chl *a* (μg Chl L^-1^)	Qy Euk (450 nm)	Qy Cyan (620 nm)
H0	0.05	1.2E+08	^∗∗∗∗∗^	^∗∗∗∗∗^	8	1.70E+05	0.35	0.55	0.23
H3	0.13	1.8E+08	^∗∗∗∗∗^	3.4E+04	14	1.80E+05	1.08	0.43	0.19
H3-RW	0.23	3.1E+08	^∗∗∗∗∗^	6.6E+03	27	1.20E+06	3.06	0.61	0.42
H4-CP	0.15	1.5E+09	^∗∗∗∗∗^	7.5E+04	200	3.80E+06	3.44	0.33	0.31
H4-IP	0.87	1.60E+09	^∗∗∗∗∗^	3.7E+04	61	6.50E+05	4.41	0.42	0.31
H3-IP	3.3	3.30E+09	4.00E+05	1.05E+04	15	2.00E+05	3.44	0.12	0.19
H4-VMP	11	4.30E+09	3.30E+06	1.47E+05	379	2.70E+06	1.64	0.40	0.19


*Average values of: bacterial secondary production (BSP), bacterial abundance (BA), picoeukaryotes abundance (PE), nanoflagellates abundance (NA), microalgae biomass (MB), microalgae abundance (MA), chlorophyll *a* (Chl *a*), photosystem II efficiency expressed as microalgae quantum yield (Qy Euk) and cyanobacteria quantum yield (Qy Cyan).*

*^∗^Seven stations were selected as follows: (a) Ho source (H0); (b) H3 source (H3); (c) H3 stream running water (H3-RW); (d) H4 connected pond (H4-CP); (e) H4 isolated pond (H4-IP); (f) H3 isolated pond (H3-IP); (g) H4 isolated pond “Virgin Mary pond” (H4-VMP). ^∗∗∗∗∗^ No cells observed.*

**FIGURE 3:**
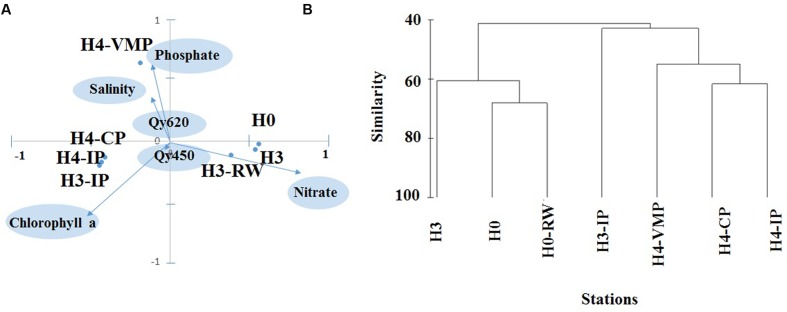
**(A)** Statistical multivariate principal component analysis to evaluate environmental similarity among stations including salinity, NO_2_^-^, PO_4_^3-^, and NO_3_^-^ concentrations, chlorophyll *a* concentration, photosynthetic efficiency of photosystem II for microalgae (Qy450) and cyanobacteria (Qy620). Stations are plotted according to their the first and second principal component (PC) scores; environmental variables are plotted according to PC1 and PC2 coefficients; because PC scores were in the range -100 to 100, they were divided by 100 and the two coordinate systems were overlaid at the same scale (ranging from -1 to 1 for both × and *y*-axis). **(B)** Group average cluster of station similarity based on microalgae abundance data across seven stations studied.

Three different groups of stations were separated by PCA according to environmental characteristics. PC1 separated H0, H3, and H3-RW sites from a second group formed by H3-CS, H3-IP, and H4-CP sites (**Figure 3A**). The most relevant environmental factors contributing to PC1 were NO_3_^-^ and chlorophyll *a* concentrations. The first group of stations were characterized by high NO_3_^-^ and low chlorophyll *a* and the opposite conditions was true for Group B. PC2 separated H4-VMP from the other two groups, based on the high salinity, high PO_4_^3-^, and low NO_3_^-^ concentrations (**Figure 3A**).

### Microalgae Community Composition

A total of 35 microalgae taxa were identified, mainly belonging to the family Bacillariophyceae (diatoms). Only five taxa were identified belonging to families Chrysophyceae (2), Euglenophyceae (1), and Cyanophyceae (2), respectively (**Supplementary Table S1**). The number of taxa did not show a trend either between systems nor stations, varying between 14 and 21 taxa (**Supplementary Table S1**). In general, the more abundant species of microalgae were also ubiquitous, detected at six or seven stations. While *Uroglena* sp. (Chrysophyceae) was the fourth most abundant organism, it was found only in H4-VMP (**Supplementary Table S1**). Some taxa were only abundant at a few sites. *Oscillatoria* sp. (Cyanophyceae) was only present in H0, H3, and H3-RW, and in the two source sites it comprised over one-third of total microalgae abundance. *Anabaena constricta* (Cyanophyceae) was found in three sites but made up 20 and 50% of total abundance in H4-CP and H3-IP, respectively.

Microalgae abundance peaked at H4-CP, followed by H4-VMP and stream waters, with lowest values in source waters. The same trend was observed for microalgae biomass except that the peak occurred at H4-VMP followed by H4-CP. At H4-VMP, the peak in biomass was due to the large diatom *Navicula decussis*. At other stations the dominating diatom was *Achnanthes lanceolata*. One-fourth of H4-VMP total microalgae abundance was due to this diatom but it was also observed in isolated ponds and the connected pond (**Supplementary Table S1**). Communities from source stations (H0, H3) and running water close to source (H3-RW) were separated from pond systems by cluster analysis at higher than 50% similarity (**Figure 3B**). Photoautotrophic picoeukaryotes were found only in ponds H3-IP and H4-VMP and their abundance was an order of magnitude higher at H4-VMP than in H3-IP (3.3 × 10^6^ and 4 × 10^5^ cells l^-1^, respectively, **Table 2**). Nanoflagellates were detected by flow cytometry across all stations except in source H0. Maximum abundance was 1.5 × 10^5^ cell l^-1^ at H4-VMP.

Mean BSP varied widely among stations. H4-VMP showed the highest mean BSP value (11.25 μg C l^-1^ h^-1^), followed by the two other isolated ponds (3.27 and 0.87 μg C l^-1^ h^-1^ for stations H3-IP and H4-IP, respectively). The lowest value was found for source water H0 (**Table 2**). BA was an order of magnitude higher in ponds compared to source and stream waters, a maximum of 4.3 × 10^9^ cells l^-1^ in H4-VMP and a minimum of 1.2 × 10^8^ cells l^-1^ at H0 source. Cellular rates of BSP were also higher in H4-VMP than at the other stations.

### BSP Response to Solar Radiation Treatments across Stations

**FIGURE 4:**
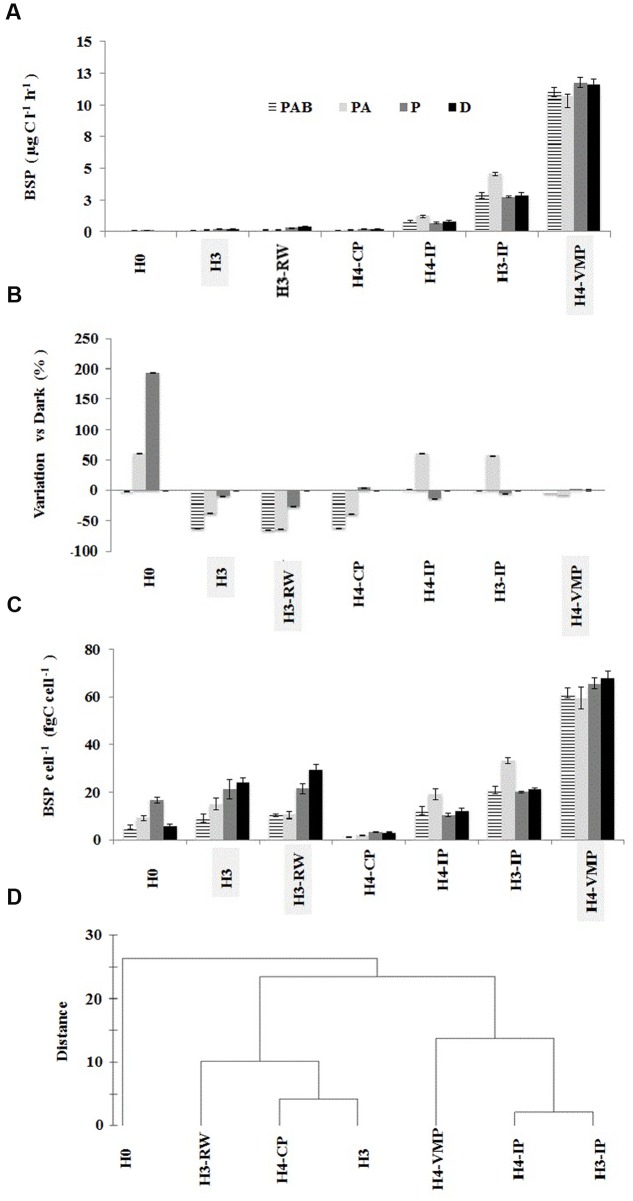
**Bacterial secondary production (BSP) experimental response to solar radiation at seven stations in Salar de Huasco.** Error bars represent standard deviation. The BSP samples from each sampling site were incubated under four different solar conditions: Full sun (PAB treatment), no UVB (PA treatment), no UV (PAR treatment), and no light exposure (dark treatment). **(A)** Average BSP under solar radiation treatments expressed as μg C l^-1^ h^-1^. **(B)** Variation of BSP as percentage of dark treatment **(C)**. BSP cell^-1^ (as proxy of growth efficiency) expressed as fg C cell^-1^ h^-1^. **(D)** Group average cluster of station similarity based on BSP response to different solar radiation treatments.

The response of BSP to solar radiation treatments showed a significant interaction (df 4; *F* = 4.6, *p* < 0.05) with the covariate distance from source (distance × treatment in covariance analysis with heterogeneous slopes). The largest BSP effect was observed in waters from H0 source (threefold enhancement after PAR exposure) while the lowest difference was observed in H4-VMP waters after PAR incubation (**Figures 4A–C**). BSP absolute response was highest at source waters (H0 and H3) and close to source running waters (H3-RW), decreasing through connected ponds and isolated ponds, reaching a lowest BSP response to solar radiation at H4-VMP (**Figures 4A–C**). BSP from H4-CP and H3 showed the same response after light exposure: PAR and DARK treatments were similar and higher than PAB and PA treatments (**Figure 4A**; **Table 3**). PAB and PA were not significantly different among them for each of the two stations. This indicates that BSP is negatively affected by UVA but less so by UVB. The same trend was also observed for H3-RW. The BSP in H0 (ground water source) was higher in PAR and PA than in the other treatments that did not differ significantly from each other. Therefore, an increasing detrimental effect of UVA and UVA + UVB is evidenced after PAR maximum enhancement over DARK conditions in H0.

**Table 3:** One-way ANOVA results for bacterial secondary production (BSP) after incubation under different solar radiation treatments.

	**Stations^∗^**	**Transformation**	**df**	**MS**	***F***	***p***	**BSP^∗∗^*Post hoc* results Tuckey**
a	H0	–	3	0.006	210.2	<0.001	P > PA > PAB = D
b	H3	TR	3	0.049	63.4	<0.001	D = P > PA > PAB
c	H3-RW	–	3	0.14	299.2	<0.001	D > P > PA = PAB
d	H4-CP	TR	3	0.06	108.5	<0.001	D = P > PA > PAB
e	H4-IP	–	3	0.52	42.28	<0.001	PA > PAB = P = D
f	H3-IP	KW-MR	3		H = 22.4	<0.001	PA > PAB = P = D
g	H4-VMP	TR	3	0.053	2.9	<0.05	PA<PAB = P = D


*Transformation indicates when the variable BSP has been root transformed (TR) to fit ANOVA homogeneity of variance and normality assumptions, or, when non-parametric Kruskal–Wallis and multiple comparison of mean ranks (MR) were applied. All differences described as (*post hoc* results) were significant at *p* < 0.05 (*post hoc* parametric Tukey-except f).*

*^∗^The response for seven sampled stations was tested independently as follows: (a) Ho source (H0); (b) H3 source (H3); (c) H3 stream running water (H3-RW); (d) H4 connected pond (H4-CP); (e) H4 isolated pond (H4-IP); (f) H3 isolated pond (H3-IP); (g) H4 isolated pond “Virgin Mary pond” (H4-VMP).*

*^∗∗^Total bacterial secondary production (BSP) and average bacterial production per cell (BSP cell^-1^) incubated under different solar radiation treatments: full sun (PAB treatment), no UVB (PA treatment), no UV, only PAR (P treatment), and no light exposure (dark treatment).*

BSP from isolated ponds H4-IP and H3-IP and H4-VMP were not significantly different between DARK, PAR, and PAB exposures (**Figure 4A**; **Table 3**) Nevertheless, BSP in PA treatment differed from other treatments, increasing in H3-IP and H4-IP and decreasing in H4-VMP waters (**Figures 4A,B**). The similarities and differences in response patterns among stations were captured by cluster analysis. Isolated pond waters grouped separately from the other stations, with H0 as the station showing the largest distance with all others.

Ground sources and running water sites (H0, H3, and H3-RW) as well as isolated ponds (H3-IP and H4-IP) had low BSP and production per cell (BSP per cell). The maximum BSP per cell was measured at H4-VMP while the lowest was in H4-CP (**Figure 4C**). BSP per cell showed the same responses to solar radiation treatments as BSP by volume in isolated ponds and H0. For H3 and running water, BSP per cell changed slightly from that of BSP, but PA and PAB were always below PAR and DARK (**Figure 4C**).

## Discussion

We reported the first BSP measurements for Salar de Huasco, as well as the first measured diurnal cycle of full spectrum solar radiation. Previously, only shortwave pyranometer estimations for total radiation (1000 W m^-2^) and heat fluxes (∼1150 W m^-2^) have been reported for Salar de Huasco area (Aceituno, 1996; De la Fuente, 2014, respectively). We also showed the relevance of distance and isolation of sampling sites from ground water sources, which contributed to spatial heterogeneity of physico-chemical conditions, microbial community composition, and BSP responses to solar radiation in Salar de Huasco.

Physico-chemical conditions have been reported to vary markedly on small spatial scales in Salar de Huasco (Risacher and Fritz, 2009). In this study, it was possible to recognize three different groups of stations based on nutrient levels (NO_3_^-^, NO_2_^-^, or PO_4_^-3^) and salinity together with chlorophyll *a* and Qy (**Figures 1C and 3A**). Source waters (H0 and H3) and close to source running waters grouped with the lowest salinities and high NO_3_^-^ concentration. Sources shared rather similar conditions as could be expected since the emerging groundwater originates from the same aquifer (Magaritz et al., 1990; Acosta and Custodio, 2008; Uribe et al., 2015). Downstream, ponds exhibited increased salinity and the depletion of NO_3_^-^, indicating evaporation and biological consumption. Therefore, the change in properties with time and distance from water sources might arise not only as a consequence of abiotic but also of biotic processes. Concentration of minerals and increased salinity through time due to evaporation, as well as mineral exchange with sediments, contribute to water properties variability (Acosta and Custodio, 2008) especially as water flows along the stream or becomes isolated from groundwater sources. Biotic processes such as the uptake of nutrients by primary producers and bacterial mats, or remineralization would impact dissolved nutrients availability. Changes in the dissolved ionic species concentrations and ratios might affect microbial organisms in their cellular ion pumps and osmolality balance creating electrochemical gradients for energy production and nutrient transport (Oren, 2013).

Two groups of locations were identified by microalgae community composition and abundance with source and “close-to-source” waters in one of them and isolated pond systems in the other. Low microalgae abundance and low chlorophyll *a* were characteristic of ground water source stations (H0, H3) as expected for recently emerged waters in contrast with downstream sites. This important biological variability between downstream sites could be associated with local conditions, in running waters (e.g., H3-RW), where the NO_3_^-^ concentration was still similar to that of source waters, we measured the highest photosynthetic efficiency for both eukaryotes and cyanobacteria. There, microalgae and bacterial biomasses were at intermediate levels. Minima of photosynthetic efficiency (Qy) were observed in source waters. In the absence of external sources of nutrients in isolated ponds, they can only sustain new production by the regeneration of nutrients through microbial coupling, as was potentially observed in H4-VMP. This site with high salinity, low NO_3_^-^, high incident solar radiation and nighttime partial freezing, had a higher microalgae biomass, higher bacterial, picoeukaryote and NA, and a BSP 1-2 orders of magnitude higher than all other stations, and showed the highest BSP/biomass ratio.

BSP variability along the sampled stations around the main lagoon of Salar de Huasco range over two orders of magnitude (**Table 2**). Unfortunately, there are no comparable estimations for salt flats wetlands or other High Altitude northern Andean locations. Nevertheless, BSP at Salar de Huasco is quite high as compared to other high altitude lakes (Sommaruga et al., 1997; Eiler and Bertilsson, 2004; Sarmento et al., 2015). BSP and bacterial numbers were higher at isolated ponds as compared to connected stations. This increase in BA and BSP in water bodies after evaporation is consistent with results from other systems. For example, in a flooding-desiccation-cycle in Silver Lake California, USA, changes in pH and total dissolved solids were inversely related to NO_3_^-^ and evaporation, which promoted an increase in BA (used as a BSP proxy) by three orders of magnitude (Navarro et al., 2009). The microbial community of isolated ponds in Salar de Huasco could be expected to be adapted to high natural solar exposure by means of gene transfer and direct selection. Biosynthesis of detoxifying enzymes or antioxidant molecules such as glutathione is among the biochemical mechanisms of tolerance reported (e.g., Wang et al., 2011; Albarracín et al., 2015a,b, 2016; Häder et al., 2015; Kurth et al., 2015). Li et al. (2014) also described high relative evolutionary rates (rERs) of microbial communities from more extreme natural environments, where the microbes inhabiting extreme habitats (acid mine drainage, saline lake, and hot spring) evolved faster than those populating benign environments (e.g., surface ocean, fresh water, and soil). This high evolutionary rate was attributed potentially to more frequent horizontal gene transfer in communities from extreme habitats.

BSP production shifts observed in Salar de Huasco responded as expected when extreme solar radiation conditions prevailed, as was observed in other aquatic systems (Sommaruga, 2001; Gasol et al., 2004; Ruiz-González et al., 2013). For well-adapted communities (long light exposure history) in the isolated ponds, the exposure to full solar radiation (PAR + UVA + UVB) did not result in production shifts, and showed the lowest response under light treatments as compared to dark condition (also observed in brines, see Pedrós-Alió et al., 2000; Benlloch et al., 2002). In contrast, significant BSP responses to PAR + UVA and full solar radiation, as compared with dark and PAR conditions, were observed in ground water sources and connected stations. For less adapted communities (recent light exposure history) as are expected in ground water sources and connected stations, the BSP response to different light treatments was stronger than that of communities with long light exposure history (Jeffrey et al., 1996a,b, 2000; Alonso-Saez et al., 2006; Hernández et al., 2007; Ruiz-González et al., 2013).

BSP responses (inhibition or enhancement) under short-term solar exposure cannot be only related to light exposure history, but also to some other extreme conditions (Rothschild and Mancinelli, 2001; Triadó-Margarit and Casamayor, 2012; Guerrero et al., 2013; Li et al., 2014). The irradiance conditions experienced by isolated pond communities in our experiments were in combination with other potentially limiting conditions. Exposing the community to PAR + UVA implies a single source of damage is removed (UVB), and productivity shifts could result from its interplay with other extreme variables such as nutrients and/or organic substrates availability (Häder et al., 2015; **Table 3**). Hence, isolated ponds (H3-IP and H4-IP) showed a productivity enhancement from dark to PAR + UVA treatment, but no enhancement from dark to PAR or PAR + UVA + UVB (**Figure 4**; **Table 3**). This would be the result, for example, if photoheterotrophic strains benefit through competitive interaction only when PAR is available and they out compete other strains when UVB is absent (Church et al., 2004).

In aquatic systems most deleterious effects on primary producers of solar radiation has been related directly to UVB damage and indirectly to UVA and some PAR photoinhibition (Helbling et al., 2001; Häder et al., 2015). Nevertheless, autotrophs, mixotrophs, and heterotrophs also develop tolerance which has been observed in isolated strains (Fernández-Zenoff et al., 2006; Flores et al., 2009; Paulino-Lima et al., 2013). In the isolated pond H4-VMP, BSP decreased only under PAR + UVA irradiance (**Table 3**). This could be due to competitive interactions with other trophic components of the system (e.g., microalgae). At H4-VMP all microbial biomass and BSP were the highest measured (**Table 2**), and BSP response to different light treatments was the lowest among all other stations. At the same time, salinity is extreme, PO_4_^3-^ concentration was high and nitrogen species reached a minimum level of all stations (**Table 1**). Microbial communities here must cope with multiple “extremes.” When one source of UV stress is removed, a new state for competitive or trophic biological interactions might be developing with very complex interactions which may be difficult to separate with only BSP, abundance or biomass measurements. For instance, Liu et al. (2014) found relationships between dominating strains and changes in biodiversity at local scales related to pH and ferrous and ferric concentrations in extreme environments. Some other factors such as conductivity and rainfall (Edwards et al., 1999), temperature and sulfide (Purcell et al., 2007), and organic carbon (Polymenakou et al., 2005) can also have an influence on the phylogenetic microbial assemblage differentiation in extreme environments.

Isolation in the complex water systems in Salar de Huasco with different physico-chemical conditions promote microbial communities which respond differently to solar radiation stress (PAR, UVA, and UVB) in a daily cycle. We propose that the adaptation of the microbial community to other extreme conditions (evaporation, nutrient limitation, temperature gradients, distance, and isolation from groundwater sources) affects the response of BSP to solar exposure directly or indirectly through biological interactions.

## Author Contributions

KH and BY conceived, organized, and wrote the paper. CD and VM obtained funding for the original project. KH, CD, WJ, LO, and FR collected data. KH, BY, LO, WJ, and EM analyzed the existing information and CD, KH, WJ, LO, VM, FR, and MH performed fieldtrips and collected biogeochemical information. KH and BY developed the paper idea and obtained additional funding for microalgae analyses. All authors have read and approved this manuscript.

## Conflict of Interest Statement

The authors declare that the research was conducted in the absence of any commercial or financial relationships that could be construed as a potential conflict of interest.
