# Supplementary material for: Microbial Activity Response to Solar Radiation across Contrasting Environmental Conditions in Salar de Huasco, Northern Chilean Altiplano
Source: Front Microbiol. 2016 Nov 22;7:1857. doi: 10.3389/fmicb.2016.01857 (PMC5118629; doi:10.3389/fmicb.2016.01857)

Supplementary Material

**Microbial activity response to solar radiation across contrasting environmental conditions in Salar de Huasco, Northern Chilean Altiplano**

Klaudia Hernández *, Beatriz Yannicelli *, Lasse Mork Olsen, Cristina Dorador, Ricardo Menschel, Verónica Molina, Francisco Remonsellez, Martha Hengst, Wade H. Jeffrey

*** Correspondence:** Corresponding Author: [klhr2009@gmail.com](mailto:klhr2009@gmail.com) ; Corresponding Author: [byannice@ceaza.cl](mailto:byannice@ceaza.cl)

**Supplementary Table 1.** Microalgae composition along Salar de Huasco samples at seven stations during November 15, 2013.

|  |  |  |  |  |  |  |  |  |
| --- | --- | --- | --- | --- | --- | --- | --- | --- |
| **Microalgae composition** | | **Microalgae abundance at Salar de Huasco** | | | | | | |
| **(Genus / Species)** | **Class** | **HO** | **H3** | **H3-RW** | **H3-IP** | **H4-CS** | **H4-IP** | **H4-VMP** |
|  |  | *cells L^-1^* | *cells L^-1^* | *cells L^-1^* | *cells L^-1^* | *cells L^-1^* | *cells L^-1^* | *cells L^-1^* |
|  |  |  |  |  |  |  |  |  |
|  |  |  |  |  |  |  |  |  |
| *Aulacoseira granulata* | Bacill. | 0 | 33100 | 0 | 0 | 0 | 0 | 0 |
| *Diploneis subovalis* | Bacill. | 0 | 1840 | 0 | 0 | 0 | 0 | 0 |
| *Fragilaria pinnata* | Bacill. | 0 | 0 | 9220 | 0 | 0 | 0 | 0 |
| *Grammatophora angulosa* | Bacill. | 0 | 440 | 0 | 0 | 0 | 0 | 0 |
| *Melosira varians* | Bacill. | 0 | 0 | 0 | 0 | 137500 | 0 | 0 |
| *Navicula pupula* | Bacill. | 0 | 0 | 0 | 1500 | 0 | 0 | 0 |
| *Pinnularia viridis* | Bacill. | 0 | 0 | 0 | 0 | 0 | 1360 | 0 |
| *Uroglena* | Chrys. | 0 | 0 | 0 | 0 | 0 | 0 | 1042333 |
| *Amphora splendida* | Bacill. | 0 | 0 | 0 | 11240 | 0 | 0 | 19000 |
| *Gomphonema gracile* | Bacill. | 0 | 0 | 0 | 0 | 0 | 1100 | 27667 |
| *Mallomonas splendens* | Chrys. | 980 | 0 | 920 | 0 | 0 | 0 | 0 |
| *Navicula rhynchocephala* | Bacill. | 5500 | 0 | 860 | 0 | 0 | 0 | 0 |
| *Phacus costatus* | Euglen | 0 | 0 | 0 | 180 | 640 | 0 | 0 |
| *Synedra acus* | Bacill. | 0 | 5020 | 5560 | 0 | 0 | 0 | 0 |
| *Synedra parasitica* | Bacill. | 380 | 0 | 0 | 2440 | 0 | 0 | 0 |
| *Anabaena constricta* | Cyan. | 0 | 10100 | 0 | 103960 | 764900 | 0 | 0 |
| *Gomphonema parvulum* | Bacill. | 1320 | 460 | 0 | 940 | 0 | 0 | 0 |
|  |  |  |  |  |  |  |  |  |

**Supplementary Table 1.** Continuation

|  |  |  |  |  |  |  |  |  |
| --- | --- | --- | --- | --- | --- | --- | --- | --- |
| **Microalgae composition** | | **Microalgae abundance at Salar de Huasco** | | | | | | |
| **(Genus /Species)** | **Class** | **HO** | **H3** | **H3-RW** | **H3-IP** | **H4-CS** | **H4-IP** | **H4-VMP** |
|  |  | *cells L^-1^* | *cells L^-1^* | *cells L^-1^* | *cells L^-1^* | *cells L^-1^* | *cells L^-1^* | *cells L^-1^* |
|  |  |  |  |  |  |  |  |  |
|  |  |  |  |  |  |  |  |  |
| *Oscillatoria sp.* | Cyan. | 68180 | 31720 | 41320 | 0 | 0 | 0 | 0 |
| *Pinnularia pinedana* | Bacill. | 820 | 500 | 0 | 0 | 10580 | 0 | 0 |
| *Rhoicosphenia curvata* | Bacill. | 0 | 920 | 1140 | 0 | 0 | 0 | 38333 |
| *Stauroneis phoenicenteron* | Bacill. | 0 | 0 | 0 | 1840 | 0 | 4240 | 32667 |
| *Cocconeis placentula* | Bacill. | 0 | 2260 | 980 | 0 | 13840 | 0 | 61667 |
| *Cyclotella glomerata* | Bacill. | 0 | 660 | 0 | 6140 | 9920 | 34900 | 0 |
| *Diatoma tenue* | Bacill. | 0 | 360 | 0 | 0 | 13280 | 32300 | 11667 |
| *Navicula decussis* | Bacill. | 1540 | 0 | 0 | 2820 | 0 | 13460 | 66000 |
| *Surirella ovalis* | Bacill. | 0 | 920 | 0 | 0 | 1720 | 2220 | 31000 |
| *Cymbella affinis* | Bacill. | 3560 | 2740 | 2780 | 0 | 10700 | 10780 | 0 |
| *Navicula salinarum* | Bacill. | 0 | 540 | 0 | 4120 | 129440 | 75420 | 76667 |
| *Diadesmis sp.* | Bacill. | 7080 | 13360 | 64340 | 0 | 339600 | 133020 | 59000 |
| *Fragilaria construens* | Bacill. | 27580 | 12420 | 1036020 | 3760 | 222100 | 0 | 33667 |
| *Synedra ulna* | Bacill. | 2940 | 5060 | 4620 | 11720 | 21160 | 10820 | 0 |
| *Achnanthes lanceolata* | Bacill. | 33100 | 12420 | 53340 | 35600 | 359580 | 236540 | 958333 |
| *Navicula radiosa* | Bacill. | 7120 | 7820 | 11040 | 4660 | 63460 | 53860 | 181000 |
| *Nitzschia acicularioides* | Bacill. | 6900 | 3260 | 10180 | 6560 | 1702780 | 43100 | 153000 |
|  |  |  |  |  |  |  |  |  |

** Seven stations were selected as follows: (a) Ho Source (H0); (b) H3 Source (H3); (c) H3 Stream running water; (H3-RW); (d) H4 Connected pond (H4-CP); (e) H4 isolated pond (H4-IP); (f) H3 Isolated pond (H3-IP); (g) H4 isolated pond "Virgin Mary pond" (H4-VMP).*


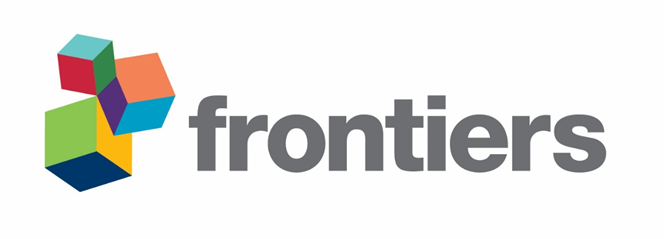

Supplement: Supplementary file 1 [file Table_1.DOCX]
