# Supplementary material for: Microbial Activity Response to Solar Radiation across Contrasting Environmental Conditions in Salar de Huasco, Northern Chilean Altiplano
Source: Front Microbiol. 2016 Nov 22;7:1857. doi: 10.3389/fmicb.2016.01857 (PMC5118629; doi:10.3389/fmicb.2016.01857)
Supplement: Supplementary file 2 [file Image_1.PDF]

Supplementary Material

Microbial activity response to solar radiation across contrasting environmental conditions in Salar de Huasco, Northern Chilean Altiplano

Klaudia Hernández *, Beatriz Yannicelli *, Lasse Mork Olsen, Cristina Dorador, Ricardo Menschel, Verónica Molina, Francisco Remonsellez, Martha Hengst, Wade H. Jeffrey

*** Correspondence: Corresponding Author: claudia.hernandez.r@unab.cl; byannice@ceaza.cl**

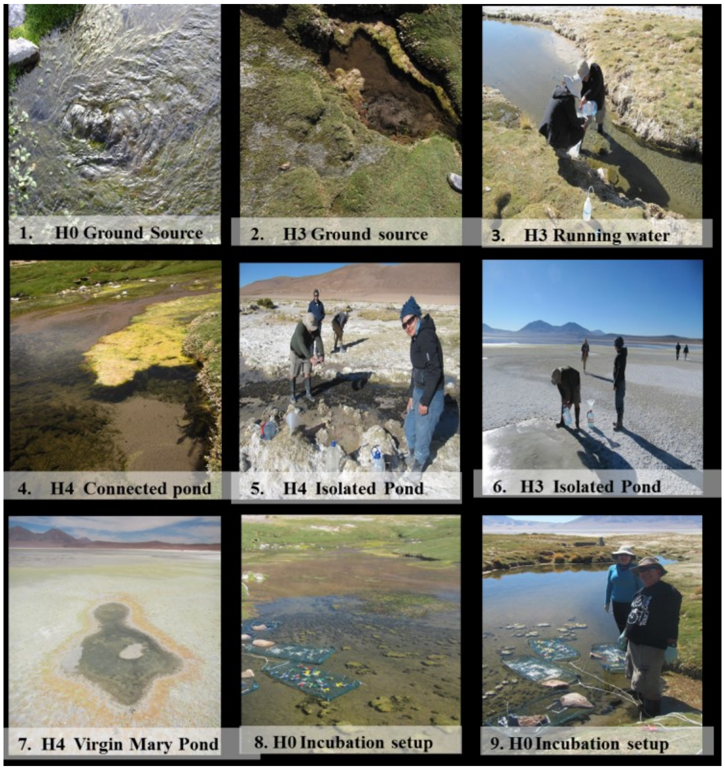

Supplementary Figure 1. Distribution and shape of sampled stations along Salar de Huasco sub-systems (Dorador et al. 2008b) considering distance and isolation from groundwater sources: (1 and 2) non-previous solar exposure, groundwater sources (stations H0 and H3), (3 and 4), solar exposed, close to ground water sources and connected ponds (stations H3-RW and H4-CP), (5-7), solar exposed isolated ponds (stations H3-IP, H4-IP, H4-VMP), (8 and 9), experimental setup for BSP response to solar radiation incubations at H0.
